# The Vaccine Control of Virus-Induced Cancers

**Published:** 1988-11

**Authors:** M. A. Epstein

**Affiliations:** Nuffield Department of Clinical Medicine, John Radcliffe Hospital, Oxford


### The Vaccine Control of Virus-Induced Cancers in Man

**Professor M. A. Epstein**
Nuffield Department of Clinical Medicine, John Radcliffe Hospital, Oxford

It would not have been acceptable 6-8 years ago to talk about virus associated cancers in man to a general medical audience, we knew what went on in mice and chickens, monkeys, cattle and cats, but to think that this had anything to do with man was taboo. In recent years all that has changed, perhaps because of studies stimulated by the advent of AIDS.

In China, three virus associated cancers account for 25% of the national cancer deaths – 700,000 a year. These are cervical cancer associated with human papilloma virus, primary liver cell cancer with hepatitis B virus and nasopharyngeal carcinoma with Epstein-Barr (EB) virus. In some areas of China where two of these virus associated cancers are common over 50% of the deaths from cancer are virus associated. World-wide it is about 20% of all cancers.

WHO has already started a 30 year prospective vaccine intervention study in the Gambia based on the MRC laboratories there, to follow the reduction of primary hepatocellular carcinoma in young adults as a result of having vaccinated the population as infants against hepatitis B virus.

There are three EB virus associated neoplastic conditions, endemic Burkitt's lymphoma, undifferentiated nasopharyngeal carcinoma and lymphomas in immunodepression – in organ graft recipients and more recently in AIDS. 98% of the world is infected with the EB virus and we live in a very delicate and efficient balance with the parasite. Once infected we have it for the rest of our lives, we develop virus neutralising antibodies which damp down the spread of the agent within the body, and specific cytotoxic T-cells which recognise the new antigens which the agent causes to be displayed on the surface of infected cells. That balance for most of us is perfect and we live with this until we die. Where we have endemic Burkitts' lymphoma and undifferentiated nasopharyngeal carcinoma there are important co-factors which are responsible for tipping this host-virus relationship into carcinogenicity by the virus. We do not know exactly how this comes about but we do know that the virus is an essential link in the chain of events that brings about the pathology. Since 1976 Professor Epstein has held the opinion that one must intervene by developing a vaccine and thereby reducing the incidence of the cancer. This, incidentally, would prove that there is a causal relationship.

There is a precedent in animals for the successful control of cancer by immunisation. Marek's disease of poultry, a herpes virus-induced disease, kills chickens with lymphomas and was of major importance in the poultry industry until vaccine against Marek's disease was introduced in the early '70s. This is the first example of an anti-viral vaccine controlling a naturally occurring cancer.

Is it worthwhile trying to do the same thing with EB virus? Burkitt's lymphoma is not really a very important problem. The numbers in the endemic areas are small and in these areas they have far worse problems. Nasopharyngeal carcinoma is a different proposition in world cancer terms. The incidence in the South of the People's Republic of China is very high and in this part of the country it is the most important cancer of men and the second most important cancer of women. For 450 million people it is the most important cancer, and it has spread with Chinese immigration into S.E. Asia, Australia, Hawaii and California. There is also a fairly high incidence right across North Africa.

The EB virus determined membrane antigen (MA) has been chosen as the basis of a vaccine. The only animal known to react with lesions after experimental infection with EB virus is the cotton top tamarin (*Seguinus oedipus oedipus*). On inoculation, these animals develop multiple malignant lymphomas in their lymph nodes, liver, gut, kidneys, suprarenals within 2 to 3 weeks – multiple malignant transformation events follow the introduction of the virus. A prototype vaccine has been produced which protects cotton top tamarins against challenge with a 100% lymphomagenic dose.

At first, great difficulty was encountered in purifying a product suitable for human use. A method using vaccinia virus incorporating the MA gene from the EB virus has been made in collaboration with Mackett in Manchester and this virus has been tested in tamarins. The original vaccine using a rather virulent vaccinia strain unsuitable for human use was protective but when the attenuated vaccination strain was tried it was not protective. The same thing was done with varicella virus using a strain already being used as a vaccine in immuno-compromised children and there have been no adverse reactions. We now have this recombinant, the problem is to decide exactly where to test it, because there is no animal model for varicella. Attenuation of the EB virus itself is not yet possible because it is a large DNA virus. We have now moved to the question of novel adjuvants. There are two new adjuvants, one developed at Uppsala and the other by Syntex research, both of which are very promising and human trials have been planned. The phase I trial, to check the responses both humoral and cellular and that it is safe, should be planned this year. After that a double blind trial is needed and it is hoped to hook this on to an ongoing prospective study which the Public Health Laboratory Service is doing in one of our Universities. This is looking at the EB virus status of new students coming up and one could concentrate on those who had not been infected as children and who were therefore at risk for infectious mononucleosis. They might therefore have the advantage of being protected against this hazard to their final examinations if vaccination were successful. After that one will go into the field as WHO is doing with Hepatitis B vaccine and hepato-cellular carcinoma. One could do it for Burkitt's lymphoma and get a result very quickly as the peak age incidence is at 6 or 7 years. And then the long term business of protecting the Southern Chinese against nasopharyngeal carcinoma would have to be tackled.

